# Kinetics of Cross-Linking Reaction of Epoxy Resin with Hydroxyapatite-Functionalized Layered Double Hydroxides

**DOI:** 10.3390/polym12051157

**Published:** 2020-05-18

**Authors:** Zohre Karami, Mohammad Reza Ganjali, Maryam Zarghami Dehaghani, Mustafa Aghazadeh, Maryam Jouyandeh, Amin Esmaeili, Sajjad Habibzadeh, Ahmad Mohaddespour, Inamuddin, Krzysztof Formela, Józef T. Haponiuk, Mohammad Reza Saeb

**Affiliations:** 1 Center of Excellence in Electrochemistry, School of Chemistry, College of Science, University of Tehran, P.O. Box 11155-4563, Tehran, Iran; zohrekarami.2013@yahoo.com (Z.K.); ganjali@ut.ac.ir (M.R.G.); mustafa.aghazadeh@gmail.com (M.A.); maryam.jouyande@gmail.com (M.J.); 2 Biosensor Research Center, Endocrinology and Metabolism Molecular-Cellular Sciences Institute, Tehran University of Medical Sciences, P. O. Box 11155-4563, Tehran, Iran; 3 School of Chemical Engineering, College of Engineering, University of Tehran, P.O. Box 11155-4563, Tehran, Iran; m.zarghami@ut.ac.ir; 4 Department of Chemical Engineering, School of Engineering Technology and Industrial Trades, College of the North Atlantic-Qatar, 24449 Arab League St, P.O. Box 24449, Doha, Qatar; amin.esmaeili-khalil-saraei@polymtl.ca; 5 Department of Chemical Engineering, Amirkabir University of Technology (Tehran Polytechnic), P.O. Box 15875-4413, Tehran, Iran; sajjad.habibzadeh@aut.ac.ir; 6 College of Engineering and Technology, American University of the Middle East, 15453 Egaila, Kuwait; ahmad.mohaddespour@mail.mcgill.ca; 7 Advanced Functional Materials Laboratory, Department of Applied Chemistry, Faculty of Engineering and Technology, Aligarh Muslim University, Aligarh 202002, India; inamuddin@zhcet.ac.in; 8 Department of Polymer Technology, Faculty of Chemistry, Gdańsk University of Technology, Gabriela Narutowicza 11/12, 80–233 Gdańsk, Poland; krzysztof.formela@pg.edu.pl; 9 Department of Resin and Additives, Institute for Color Science and Technology, P.O. Box 16765-654, Tehran, Iran

**Keywords:** *cure index*, epoxy, layered double hydroxide (LDH), hydroxyapatite, curing kinetics, isoconversional methods

## Abstract

The cure kinetics analysis of thermoset polymer composites gives useful information about their properties. In this work, two types of layered double hydroxide (LDH) consisting of Mg^2+^ and Zn^2+^ as divalent metal ions and CO_3_^2−^ as an anion intercalating agent were synthesized and functionalized with hydroxyapatite (HA) to make a potential thermal resistant nanocomposite. The curing potential of the synthesized nanoplatelets in the epoxy resin was then studied, both qualitatively and quantitatively, in terms of the *Cure Index* as well as using isoconversional methods, working on the basis of nonisothermal differential scanning calorimetry (DSC) data. Fourier transform infrared spectroscopy (FTIR) was used along with X-ray diffraction (XRD) and thermogravimetric analysis (TGA) to characterize the obtained LDH structures. The FTIR band at 3542 cm^−1^ corresponded to the O–H stretching vibration of the interlayer water molecules, while the weak band observed at 1640 cm^−1^ was attributed to the bending vibration of the H–O of the interlayer water. The characteristic band of carbonated hydroxyapatite was observed at 1456 cm^−1^. In the XRD patterns, the well-defined (00l) reflections, i.e., (003), (006), and (110), supported LDH basal reflections. Nanocomposites prepared at 0.1 wt % were examined for curing potential by the *Cure Index* as a qualitative criterion that elucidated a *Poor* cure state for epoxy/LDH nanocomposites. Moreover, the curing kinetics parameters including the activation energy (*E*_α_), reaction order, and the frequency factor were computed using the *Friedman* and Kissinger–Akahira–Sunose (*KAS*) isoconversional methods. The evolution of *E*_α_ confirmed the inhibitory role of the LDH in the crosslinking reactions. The average value of *E*_α_ for the neat epoxy was 54.37 kJ/mol based on the *KAS* method, whereas the average values were 59.94 and 59.05 kJ/mol for the epoxy containing Zn-Al-CO_3_-HA and Mg Zn-Al-CO_3_-HA, respectively. Overall, it was concluded that the developed LDH structures hindered the epoxy curing reactions.

## 1. Introduction

Epoxy resins, well-known thermosetting polymers, have been extensively used as versatile coatings and adhesives due to their promising features such as corrosion resistance, appropriate mechanical strength, chemical stability, and desirable adhesion to different surfaces/substrates [1,2,3]. However, achieving high mechanical properties and the thermal stability of thermosetting epoxy for high performance coatings has been a matter of controversy [4,5]. The incorporation of nanoparticles has been recognized as the most commonly practiced method for enhancing the ultimate properties of epoxy coatings [6].

Layered double hydroxide (LDH) falls into the category of lamellar solid materials composed of stacking brucite-like layers of metal hydroxide possessing positive charges [7,8]. These positively-charged sheets are exfoliated and neutralized by anions located between the layers as well as water molecules [9]. The advantages of cost-effectiveness, biocompatibility, pH dependent solubility, ion-exchange capability, and ability to adjusting the properties through the variation of anions and metal hydrides introduced LDHs as promising candidates for medical, industrial, and environmental applications [10,11]. The introduction of LDH into the epoxy matrix as reinforcement has also attained considerable attention due to the nontoxicity, layered structure, and high content of water [12].

Hydroxyapatite (HA) is a kind of phosphorus-containing ceramic biomaterial contributing to diver advanced materials and systems [13,14]. The combined incorporation of LDHs and ceramic materials such as HA in the form of HA-functionalized LDH in the epoxy matrix can effectively improve the mechanical strength and thermal stability of epoxy matrix for high performance applications such as flame retardant coatings and hard tissue implants for bone repair [15,16].

The ultimate properties of cross-linkable polymers such as epoxy thermoset resin strongly depend on the formation of a completely cured network [17]. In terms of thermosetting epoxy resins, their curability in the presence of any additives and nanofillers has been key for research [18,19]. Therefore, the upward trend for epoxy nanocomposite exploitation necessitates research on the structure–properties correlation [20]. Understanding the curing process for epoxy nanocomposites is a big step for optimizing the properties. The curing mechanism and kinetics parameters can be determined by computations on nonisothermal scanning calorimetry (DSC) data.

In our previous studies, the curing process for epoxy in the presence of the Zn-Al and Mg-Al LDHs with NO_3_^−^ and CO_3_^2−^ anions was evaluated qualitatively with the *Cure Index* (*CI*) [21,22,23,24]. As mentioned earlier, considering the importance of the 3D network formed during curing process in epoxy nanocomposite containing the functionalized LDHs, the focus of this research is placed on the evaluation of the curing process for epoxy nanocomposites. The surface functionalization of carbonate intercalated Zn-Al and Mg-Al LDHs with HA was performed and the resulting structures were fully characterized by Fourier-transform infrared spectroscopy (FTIR), X-ray diffraction (XRD), and thermogravimetric analysis (TGA). Finally, the curing assessment of epoxy nanocomposites was investigated by nonisothermal DSC analysis, considering the important role of HA-functionalized LDH in the curability of epoxy resin. Besides, the curing kinetics of the epoxy/LDHs nanocomposites were modeled by isoconversional methods including differential *Friedman* and integral Kissinger–Akahira–Sunose (*KAS*) methods.

## 2. Materials and Methods

### 2.1. Materials

The chemicals magnesium hexahydrate nitrate (Mg(NO_3_)_2_·6H_2_O, 99%), zinc hexahydrate nitrate (Zn(NO_3_)_2_·6H_2_O, 98%), aluminum nitrate nonahydrate (Al(NO_3_)_3_·9H_2_O, 99.9%), nitric acid (HNO_3_), sodium hydroxide (NaOH), hydroxyapatite (Ca_10_(PO_4_)_6_(OH)_2_) and sodium carbonate (Na_2_CO_3_) were used for synthesizing LDHs and purchased from Sigma Aldrich Co., Steinheim am Albuch, Germany. A diglycidyl ether of bisphenol A epoxy resin (epoxide equivalent weight of 174 g/eq) was used as the main matrix of the nanocomposite and cured with triethylenetetramine (TETA) hardener that was acquired from Sigma Aldrich Co., Milan, Italy.

### 2.2. Synthesis of HA-Functionalized LDHs

#### 2.2.1. Synthesis of Mg-Al-CO_3_-HA LDH

Mg-Al-LDH was synthesized by using three different types of prepared solution denoted as A, B, and C. For the preparation of the solution A, Mg(NO_3_)_2_·6H_2_O (0.008 mol, 2.05 g) and Al(NO_3_)_3_·9H_2_O (0.002 mol, 0.75 g) with an Mg/Al molar ratio of 4:1 were dissolved in 25 mL of deionized water. Next, NaOH (0.006 mol, 0.24 g) and Na_2_CO_3_ (0.005 mol, 0.53 g) were dissolved in 25 mL of deionized H_2_O for the preparation of the solution B. The solution C, which was, in fact, a dispersion, contained only 0.002 mol (2.008 g) hydroxyapatite in 25 mL of deionized water. In the synthesis procedure, the solution A was added into the solution B drop by drop at a rate of 6.0 mL min^−1^ under stirring. Subsequently, the resulting solution was mixed with the solution C. During the reaction, the pH was fixed at 10.5 using a 1.0 M NaOH solution. The obtained solution was poured into a Teflon-lined autoclave vessel and heated for 24 h at 170 °C. The resulting Mg-Al-CO_3_-HA LDH product was filtered and washed repeatedly with deionized H_2_O and finally dried at 60 °C for 12 h.

#### 2.2.2. Synthesis of Zn-Al-CO_3_-HA LDH

Similar to the preparation of Mg-Al-CO_3_-HA LDH, for the synthesis of the Zn-Al-CO_3_-HA LDH, 3 solutions denoted as B, C, and D were used. For this purpose, Zn(NO_3_)_2_·6H_2_O (0.004 mol, 2.12 g) was mixed at a 2:1 molar ratio with Al(NO_3_)_3_·9H_2_O (0.002, 1.5 g) and then added into 20 mL of deionized water for the preparation of solution D. The obtained solution (D) was dropped in 20 mL of the solution B at the rate of 6.0 mL min^−1^, which was then was added to solution C at a pH of 10. The mixture of the solutions B, C, and D was then heated at 60 °C for 24 h in a Teflon-lined autoclave vessel. The prepared Zn-Al-CO_3_-HA LDH product was separated by filtration and washed several times using double distilled water and dried at 60 °C for 12 h in air.

### 2.3. Preparation of Epoxy/LDH Nanocomposites

In this work, epoxy/LDH nanocomposites, containing 0.1 wt % Zn-Al-CO_3_-HA or Mg-Al-CO_3_-HA LDHs, were prepared via the solution method. First, LDHs were sonicated in chloroform for 30 min in an ice bath, then the resulting LDH dispersion was mixed with the epoxy resin under stirring at room temperature until the evaporation of the chloroform. Finally, just before the DSC test, the stoichiometric content of the TETA curing agent (100:14) was added into the epoxy resin and was thoroughly mixed. The formulation of prepared epoxy samples is listed in Table 1.

### 2.4. Characterization Methods

The crystalline structure of the synthesized LDH was examined by X-ray diffraction (XRD) analysis and the calculated basal spacing and intermetallic distance of the synthesized structures. The XRD patterns of the synthesized LDHs were obtained using a diffractometer (Bruker AXS D8 Advance, East Cheryl Parkway, Madison, WI, USA) with CuKα radiation at 40 kV and 30 mA. The XRD data were collected in the 2theta range of 2°–70° with steps of 0.05.

The chemical structures of the synthesized LDHs were evaluated with a Fourier transform infrared (FTIR, Bruker Vector 22 IR instrument, Manasquan, NJ, USA) instrument. Infrared spectra were reported using a Bruker Fourier transform infrared spectrometer within the wavenumber range of 4000 to 400 cm^−1^ using the KBr dilution technique (1.5 *w/w* %).

Thermogravimetric analysis (TGA) was conducted on a Seiko Extar 6300 (Chiba, Japan) in order to analyze the thermal stability of the LDH nanosheets. The thermogram curves were obtained in the temperature range of 30–900 °C under a heating rate of 10 °C/min in a nitrogen atmosphere with a flow rate of 50 mL/min.

The nonisothermal differential scanning calorimetry (DSC) analyses were performed with a DSC Q200 model, TA instrument (New Castle, DE, USA) from −50 to 250 °C at a heating rate (β) of 2, 5, 7, or 10 °C min^−1^ in a nitrogen atmosphere with flow rates of 50 mL/min in order to study the curing process for the epoxy/LDHs nanocomposites. It is assumed that the exothermic curing reaction was completed by a change in the DSC curve to the main baseline. The total heat of curing reaction was determined by the calculation of the total area under the exothermic DSC curve.

## 3. Results and Discussion

### 3.1. Characterization of LDHs

**Figure 1:**
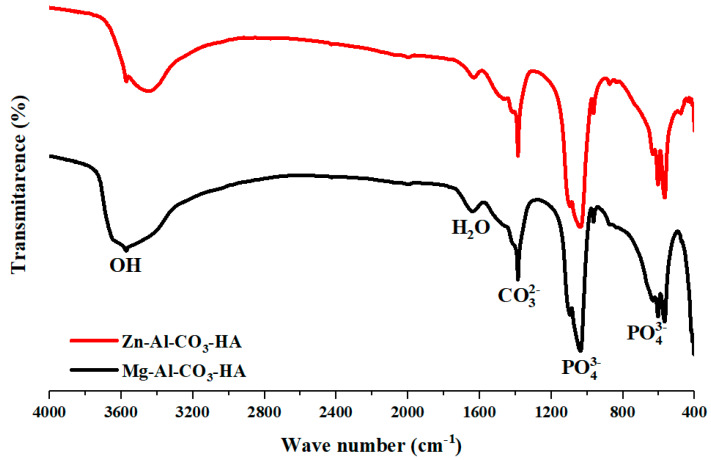
FTIR spectra of Zn-Al-CO_3_-hydroxyapatite (HA) and Mg-Al-CO_3_-HA layered double hydroxide (LDHs).

The FTIR spectra of the synthesized LDH samples are presented in Figure 1. As shown in Figure 1, the FTIR analyses display similar patterns for Zn-Al-CO_3_-HA and Mg-Al-CO_3_-HA LDHs. For both samples, the IR band at about 3542 cm^−1^ corresponds to the O–H stretching vibration of the interlayer water molecules and the –OH groups in the LDH layers [24,25]. The weak band observed at 1640 cm^−1^ can be attributed to the bending vibration of H-O of the interlayer water [26,27]. Moreover, the characteristic bands of the carbonate ion are present at 1456 cm^−1^, related to carbonated hydroxyapatite. Moreover, the stretching vibration of the carbonate ions in the interlayers of the LDHs is observed at 1383 cm^−1^ [27,28]. At the low wavenumbers, the bands at 425 and 564 cm^−1^ can be ascribed to the presence of Mg–O and Al–O bonds related to the LDH structure [25,26]. Furthermore, the presence of the bands at 1093, 1033, 991, and 633 cm^−1^ is related to the phosphate groups of hydroxyapatite [29]. The IR data clearly verified the structure of Zn-Al-CO_3_-HA and Mg-Al-CO_3_-HA LDHs.

**Figure 2:**
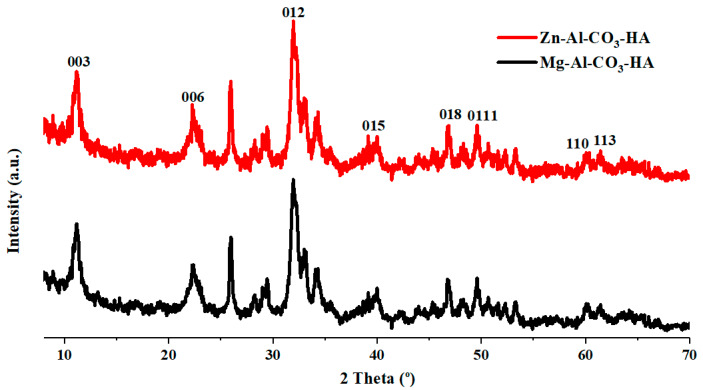
XRD analysis of Zn-Al-CO3-HA and Mg-Al-CO3-HA LDHs.

Figure 2 represents the XRD patterns of the synthesized Zn-Al-CO_3_-HA and Mg-Al-CO_3_-HA LDHs. As seen in Figure 2, the XRD analysis shows similar patterns for both LDH samples. In these patterns, well-defined (00l) reflections, such as (003), (006), and (110), are observed that are in a good agreement with the reported LDH basal reflections in the literature [24,25]. The basal spacing (d003) of Zn-Al-CO_3_-HA and Mg-Al-CO_3_-HA LDHs was calculated to be 7.91 Å and 7.81 Å compared to the values of 7.47 Å and 7.82 Å reported for Zn-Al-CO_3_ and Mg-Al-CO_3_ LDHs, respectively [22,24]. According to the XRD results, the distance between the LDH layers in the presence of the phosphate anion did not increase, implying that the phosphate anion was not replaced between the layers. The strong interaction of intercalated carbonate ions with the hydroxyl groups of LDH layers leads to the absence of ion exchange between the carbonate and phosphate anions in the gallery space [30]. It seems that the negative charge of PO_4_^3−^ causes the electrostatic absorption of anions to the outer surfaces of LDH sheets [31]. The unit cell parameters *a* and *c* were determined from the positions of these reflections, which are presented in Table 2, where a = 2d_110_ and c = 3d_003_.

**Figure 3:**
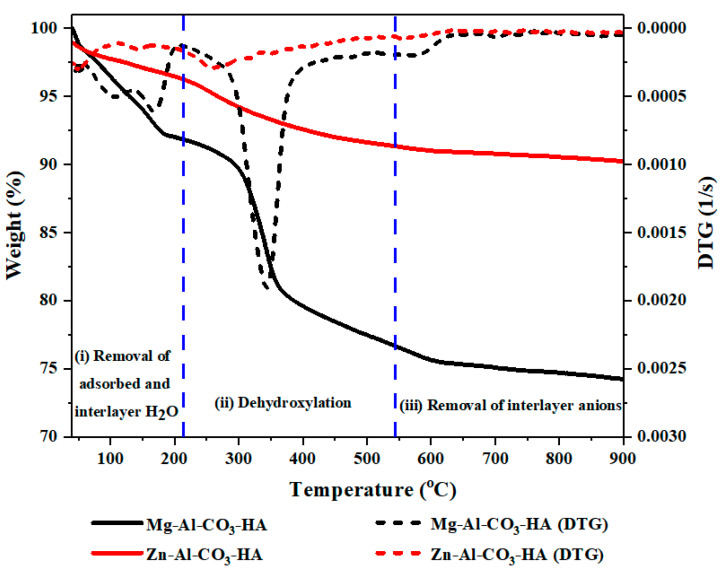
Thermogravimetric (TGA)/DTG analysis of Zn-Al-CO_3_-HA and Mg-Al-CO_3_-HA LDHs.

Figure 3 shows the TGA curves for the prepared Zn-Al-CO_3_-HA and Mg-Al-CO_3_-HA LDHs. The thermal decomposition of the LDHs appeared to undergo a third-step mass loss in the TGA curves, as shown in Figure 3, and correspondingly, endothermic peaks are present on the DTG curve. The total weight loss of the Mg-Al-CO_3_-HA is found to be 25.92 wt %, as seen in the TGA curve (Figure 3). On the DTG curve, the first step is seen at temperatures lower than 200 °C, and a weight loss of about 3.8% is observed due to the evaporation of adsorbed water and of 4.2% of the total mass, which is related to the removal of the interlayer water. The second step located within the temperatures range of 200–600 °C with a sharp peak at 346 °C can be attributed to the dehydroxylation of the LDH structures with a relatively large weight loss (i.e., 16.4%) [32]. The last main weight loss (i.e., 1.52%), located within the temperature range of 600–900 °C, is also related to the removal of the interlayer anions and decarbonation [29,32], as presented in the TGA and derivative of TGA (DTG) curves (Figure 3). Comparing the total weight loss of Mg-Al-CO_3_-HA LDH (25.92 wt %) with the corresponding value for Mg-Al-CO_3_ LDH (54.95 wt %) at 900 °C indicates the high thermal stability of LDH via HA surface functionalization [24]. As can be seen in Figure 3, in case of Zn-Al-CO3-HA LDH, there is a significant improvement in the thermal stability, while the weight loss dropped dramatically. The total weight loss of Zn-Al-CO_3_-HA is 9.89 wt %, which is much lower than the total weight loss reported for Zn-Al-CO_3_-LDH (38.63 wt %) [22]. Finally, it can be concluded that the presence of phosphorus compounds in LDHs can significantly improve the thermal stability of the structures.

### 3.2. Cure Analysis

#### 3.2.1. Qualitative Description by the Cure Index

**Figure 4:**
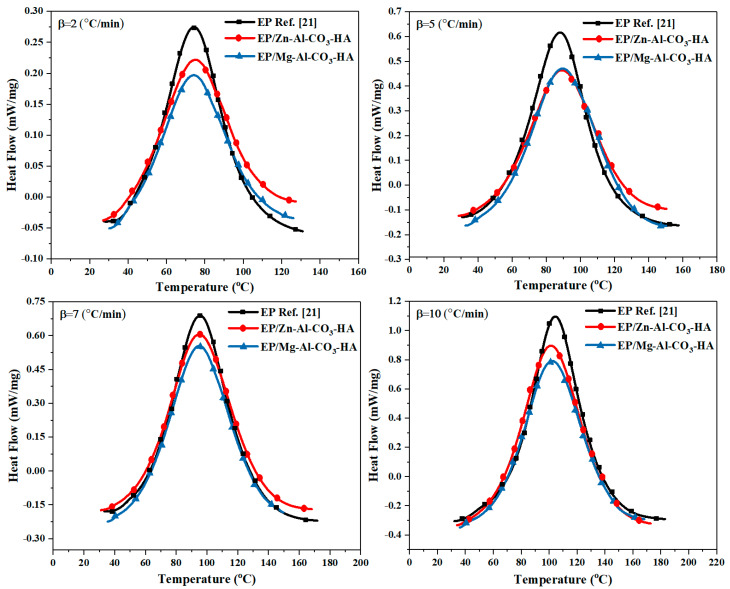
Dynamic differential scanning calorimetry (DSC) thermograms of EP [21], EP/Zn-Al-CO_3_-HA, and EP/Mg-Al-CO_3_-HA at different heating rates.

Considering the key role of nanoparticles in the curing potential of epoxy nanocomposites, the curing reaction of epoxy resin containing 0.1 wt % of Zn-Al-CO_3_-HA or Mg-Al-CO_3_-HA LDHs was evaluated via nonisothermal DSC measurements at heating rates of 2, 5, 7, and 10 °C/min (Figure 4). As shown in Figure 4, the observation of an exothermic peak for neat epoxy as the referenced sample [21] and its nanocomposites containing 0.1 wt % Zn-Al-CO_3_-HA or Mg-Al-CO_3_-HA LDHs in all heating rates revealed the domination of the ring opening reaction in the curing mechanism and single step curing kinetics [33].

The qualitative curing study of epoxy/LDHs nanocomposites was performed by the total protocols for the curing of thermosets and assessed by the *Cure Index* (*CI*). The *CI* can be obtained using the following equations [34]:

(1) ΔH*=ΔHCΔHRef

(2) ΔT*=ΔTCΔTRef

(3) $$CI=\Delta H^{*}\times \Delta T^{*}$$

The cure parameters for the EP/Zn-Al-CO_3_-HA and EP/Mg-Al-CO_3_-HA nanocomposites at the different heating rates of 2, 5, 7, and 10 °C/min are extracted from Figure 4 and represented in Table 3. The onset and endset curing temperatures (*T_onset_* and *T_endset_*), the total cure temperature (Δ*T*= *T_endset_* − *T_onset_*), the peak temperature of curing (*T_p_*), the total heat release during the curing reaction (Δ*H*_∞_), and the dimensionless parameters of ΔT*, ΔH*, and the *CI* are listed in Table 3. As shown in Table 3, the Δ*H_∞_* for the epoxy/LDH nanocomposites was significantly reduced at all heating rates. As listed in Table 3, the onset temperature of curing for all samples was increased by the enhancement of heating rates due to the increase in the energy of the system. A rapid rise in temperature did not provide enough time to start the curing in the systems, and the reaction began at a higher temperature. Moreover, at a constant heating rate, an increase in *T_onset_* was observed for the epoxy containing Mg-Al-CO_3_-HA because of the hindrance effect of the LDH layers. However, for the EP/Zn-Al-CO_3_-HA nanocomposite, *T_onset_* was slightly reduced thanks to the catalytic role of Zn^2+^ in the curing reaction. Similar to the changing trend of *T_onset_*, increases in *T_p_* and Δ*T* were observed for all samples with increases in heating rates, which indicates that the curing reaction was facilitated because of the enhancement of the kinetic energy and number of collisions in the epoxy/amine systems per unit volume. On the other hand, the Δ*T* values for the epoxy nanocomposites compared to the neat epoxy were decreased at a constant heating rate, due to the restriction of the curing reaction in the presence of LDHs in the epoxy resin. Generally, the obtained results show that introduction of HA-functionalized LDHs into the epoxy resin hindered the ring-opening of the epoxy, which led to a significant reduction in the amount of Δ*H_∞_* compared to neat epoxy during the curing reaction.

**Figure 5:**
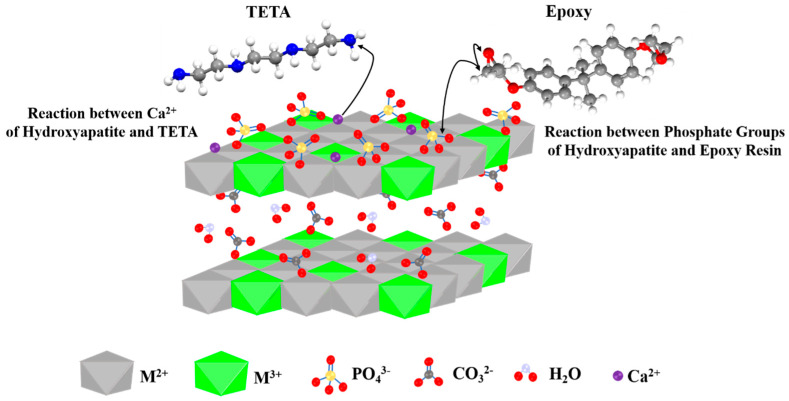
Possible reactions between hydroxyapatite (HA)-functionalized LDHs with epoxy resin and TETA curing agent.

The phosphate groups of the hydroxyapatite on the surface of LDHs could interact with the epoxide rings and participate in the curing reaction (Figure 5). By contrast, as shown schematically in Figure 5, the Ca^2+^ of the hydroxyapatite could interact with the hydroxyl groups on the LDH surface and react with the amine groups of triethylenetetramine (TETA), leading to the consumption of the curing agent in the epoxy/amine system. As the Δ*H_∞_* values represented, in competition reactions between phosphate/epoxy and calcium/amine, the consumption of the curing agent had a more apparent role, which is reflected in the less cross-linked network (lower Δ*H_∞_* values).

**Figure 6:**
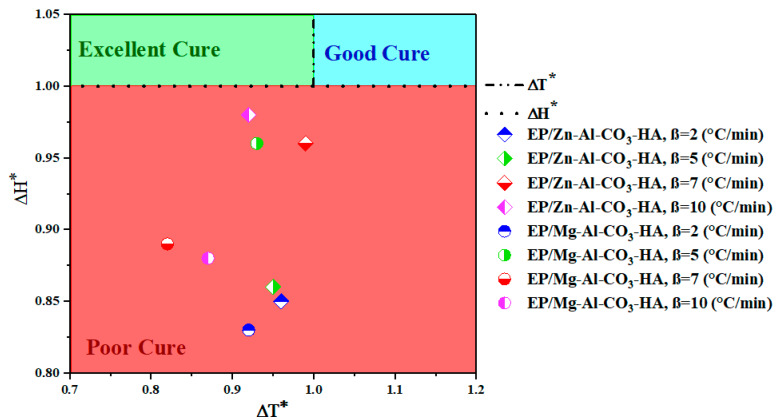
Cure state of the EP/Zn-Al-CO_3_-HA and EP/Mg-Al-CO_3_-HA nanocomposites at different heating rates.

The *CI* values for the epoxy/LDH nanocomposites are shown in Figure 6 with three different colors. According to the definition of *CI*, the green color shows an *Excellent* cure zone ($\Delta T^{*}<CI<\Delta H^{*}$), *Good* curing is demonstrated in blue ($CI>\Delta H^{*}$), and the red zone shows a *Poor* curing state ($CI<\Delta T^{*}$) for epoxy nanocomposites [35]. As demonstrated in Figure 6, the cure state for epoxy nanocomposites containing Zn-Al-CO_3_-HA and Mg-Al-CO_3_-HA LDHs are shifted to a *Poor* curing state and placed in the red region. For epoxy nanocomposites containing Mg-Al-CO_3_-HA, the cure potential is labeled as *Poor* for all heating rates, which is due to the consumption of the amine curing agent by carbonate ions. However, the existence of the phosphate groups on the HA-functionalized Mg-Al-CO_3_ LDHs shows low compensation of Δ*H_∞_* compared to the pristine LDHs [24]. For epoxy/Zn-Al-CO_3_-HA, a reduced heat release is also observed at all heating rates, which is contrary to the observations for the epoxy nanocomposite containing Zn-Al-CO_3_ [22]. As reported for epoxy/ Zn-Al-CO_3_, the cure state of these systems is located in the green area (*Excellent CI*) at a heating rate of 5 and 7 °C/min because of the acid Lewis effect of Zn^2+^ in the curing reaction, while these results are inconsistent with the cure potential estimated for the epoxy/Zn-Al-CO_3_-HA nanocomposites [22]. It seems that the carbonate ions on the surface of the Zn-Al-CO_3_-HA LDH prevent the reaction between the epoxide rings of the resin and amine groups of the curing agent molecules by engaging amine groups with the surface of the LDH.

#### 3.2.2. Qualitative Cure Assessment by Isoconversional Methods

**Figure 7:**
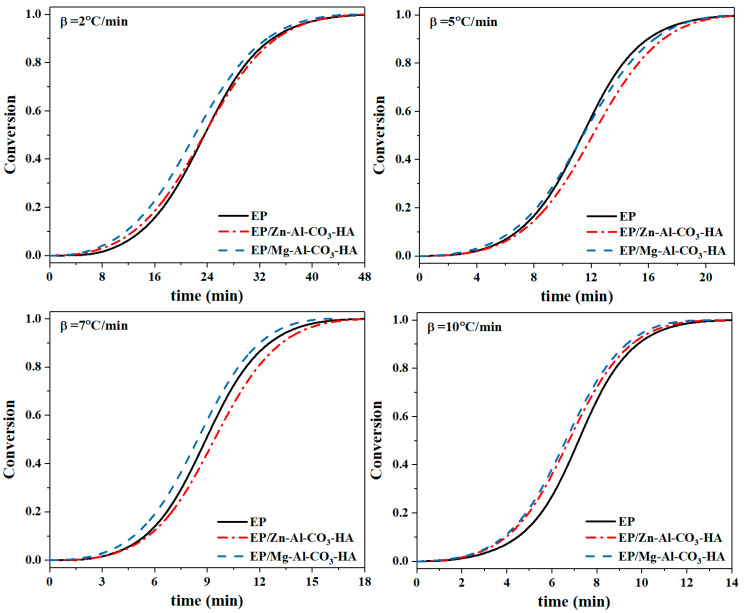
The α-time curves for the EP, EP/Zn-Al-CO_3_-HA, and EP/Mg-Al-CO_3_-HA nanocomposites at heating rates of 2, 5, 7, and 10 °C∙min^−1.^

The fractional extent of the cure reaction (α), which is directly related to the heat of curing, can be obtained from the following equation [36,37]:

(4) $$\alpha =\frac{\Delta H_{T}}{\Delta H_{\infty }}$$

where Δ*H_∞_* represents the total heat release during the curing reaction and Δ*H_T_* denotes the curing enthalpy up to temperature *T*. The fractional extent of the curing conversion as a function of the reaction temperature is presented in Figure 7. The sigmoidal shape of the presented curves in Figure 7 elucidate the autocatalytic mechanism of the curing reaction for all samples due to the generation of hydroxyl groups in the curing moiety [38]. According to the conversion–time curves, increasing the heating rates from 2 to 10 °C∙min^−1^ accelerates the epoxide ring-opening curing reaction. By enhancing the molecules’ motion and raising the number of collisions in the epoxy/amine systems due to the higher kinetic energy per molecule at higher heating rates, the curing reaction is completed in a shorter time for all samples [39]. As demonstrated in Figure 7, the epoxy nanocomposite consisting of Mg-Al-CO_3_-HA showed a higher curing rate at all heating rates and took less time for the reaction with respect to the two other samples, which is due to the incomplete curing of the EP/Mg-Al-CO_3_-HA. In the case of the EP/Zn-Al-CO_3_-HA, as reported in Table 3, the lower value for the heat release during the curing reaction for the epoxy nanocomposite containing the Zn-Al-CO_3_-HA represents incomplete curing compared to the neat epoxy, but the presence of Zn^2+^ in the curing moiety led to the continuation of the reaction for more time, although the presence of carbonate ions and phosphate groups on the LDH surfaces prevented the positive role of these cations in improving the epoxide ring opening reaction.

**Figure 8:**
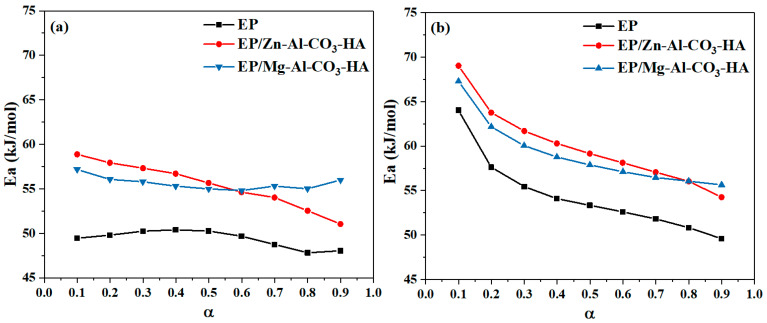
Estimation of the activation energy for the EP, EP/Zn-Al-CO_3_-HA, and EP/Mg-Al-CO_3_-HA nanocomposites: (**a**) differential *Friedman* and (**b**) integral Kissinger–Akahira–Sunose (*KAS*) models.

Isoconversional method (model-free) approaches were used for evaluating the kinetic parameters of epoxy in the presence of the Zn-Al-CO_3_-HA and Mg-Al-CO_3_-HA LDHs. In the isoconversional methods, it is assumed that the curing reaction rate at a specified α is only temperature dependent [40,41]. The differential *Friedman* method and integral *KAS* model were selected to obtain nonisothermal kinetics parameters [42,43]. The equations of the *Friedman* and *KAS* models are described in Appendix A (Appendix A.1 and Appendix A.2). The activation energies (*E_α_*) for neat epoxy and its nanocomposites as a function of the extent of curing from both the *Friedman* and *KAS* models are shown in Figure 8. As demonstrated in Figure 8, the presence of LDHs in epoxy resin leads to an increase in *E_α_* values compared to neat epoxy. In other words, the viscosity increase of the epoxy nanocomposites containing platelet-like LDHs prevents the amine molecules from accessing the epoxide rings in the curing moiety and, consequently, raises the activation energy *E_α_*of the curing reaction of the nanocomposites [44,45]. Moreover, the incomplete curing of the epoxy nanocomposite in the presence of the HA-functionalized LDHs is one of the reasons for increasing the activation energy of epoxy/amine systems. Moreover, the reduction in *E_α_,* calculated by the KAS model, increasing at higher conversions (α > 0.2), confirms the autocatalytic mechanism of the curing reaction [46].

The average values of *E_α_* over the whole range of α based on the *Friedman* and *KAS* methods are summarized in Table 4. As reported in Table 4, the *E_α_* values calculated by both the *Friedman* and *KAS* models for the epoxy nanocomposites are generally higher with respect to those of the neat epoxy. This is mainly due to the viscosity increase in the epoxy/amine systems in the presence of LDHs. In addition, determining the reaction model and degree of the curing reaction by the *Friedman* and *Málek* methods is fully explained in Appendix B and Appendix C, respectively. According to the verification of the autocatalytic curing reaction by the *Málek* method (Appendix B.2 in Appendix B), the degrees of the autocatalytic and noncatalytic reactions (*m* and *n*) and the frequency factor (ln *A*), which are explained in Appendix C, are represented in Table 4. As shown in Table 4, the m values, which show the autocatalytic order of the curing reaction, decreased in the epoxy/LDHs nanocomposites. For both epoxy nanocomposites, the hindrance effect of the HA-functionalized LDHs in the curability of the systems leads to a reduction in the autocatalytic degree of the curing reaction, and m values calculated by the *Friedman* method are reduced from 0.44 for epoxy resin to 0.21 and 0.20 for the epoxy nanocomposites containing Zn-Al-CO_3_-HA and Mg-Al-CO_3_-HA, respectively. On the other hand, the frequency factor, which indicates the number of molecular collisions in the epoxy/amine systems, increased in the presence of the HA-functionalized LDHs. According to the results, due to the competition of carbonate and phosphate, the reaction between epoxy and amine was hindered as reflected in the lower values of *m* and *n*.

**Figure 9:**
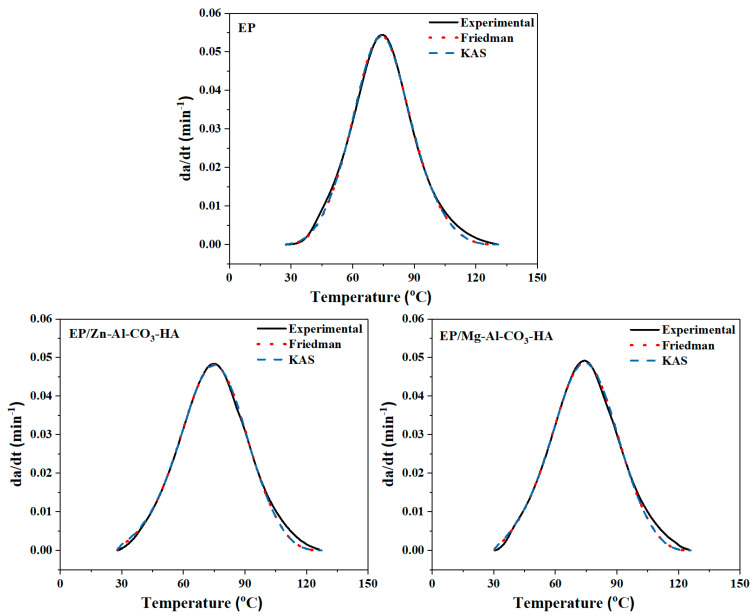
Comparison of the experimental curing rates with the values obtained from the kinetic models (*Friedman* and *KAS*).

In order to verify the curing kinetic parameters of the epoxy/amine systems, the obtained curing rates (*dα/dt*) from both the isoconversional *Friedman* and *KAS* models compared with the experimental data are shown in Figure 9. As can be seen in Figure 9, the results of the theoretical models for the epoxy/LDH nanocomposites are fully consistent with the laboratory results.

## 4. Conclusions

In this work, hydroxyapatite functionalized LDHs were synthesized and characterized by FTIR, XRD, and TGA/DTG analyses. Moreover, the curing reaction for epoxy nanocomposites containing 0.1 wt % HA-functionalized LDHs was studied using the nonisothermal DSC. The FTIR analysis confirmed the presence of phosphate groups of hydroxyapatites. Besides, the TGA/DTG analysis showed a significant improvement in the thermal stability of the HA-functionalized LDHs. The obtained results demonstrate a total weight loss of 9.89 and 25.92 wt % for Zn-Al-CO_3_-HA and Mg -Al-CO_3_-HA LDHs, respectively, which indicates a remarkable reduction in thermal decomposition compared to pristine LDHs. Investigating the cure potential of the epoxy nanocomposites in the presence of HA-functionalized LDHs elucidates that the curing reaction is hindered in the presence of LDHs in the curing moiety, and the *CI* criteria shifted to the *Poor* area. Moreover, the *E_α_* values, as a function of *α* for epoxy/LDH nanocomposites, increased for higher *α*, which confirms the inhibitory role of the LDHs in the curing reaction. For example, the average value of *E_α_* for the neat epoxy is determined to be 49.38 kJ/mol based on the *Friedman* method, compared to 55.41 and 55.61 kJ/mol for epoxy containing Zn-Al-CO_3_-HA and Mg-Al-CO_3_-HA LDHs, respectively. Based on the results achieved in this work, it can be deduced that a *Poor* cure state in the studied system takes place regardless of the heating rate and the type of intercalating ions. This would be considered as the main reason for the inadequate thermal and mechanical properties observed in the assigned systems. In other words, cross-linking analysis makes it possible to judge about the changes in the properties of thermoset nanocomposites.

## Figures and Tables

**Table 1:** Weight compositions of epoxy samples.

Sample	Nanoparticle	Content (wt %)
Ep	-	0.0
Ep/Zn-Al-CO_3_-HA	Zn-Al-CO_3_-HA LDH	0.1
Ep/Mg-Al-CO_3_-HA	Mg-Al-CO_3_-HA LDH	0.1

**Table 2:** Crystallographic parameters of the samples.

Sample	2θ of Main Peaks			Basal Spacing (C/3) (Å)	Intermetallic Distance (2a) (Å)
	(003)	(006)	(110)		
**Zn-Al-CO_3_-HA**	11.17	22.12	60.28	7.91	3.06
**Mg-Al-CO_3_-HA**	11.31	22.38	60.28	7.81	3.08

**Table 3:** Cure characteristics of the prepared epoxy nanocomposite as a function of the heating rate.

Designation	β (°C/min)	*T_onset_* (°C)	*T_endset_* (°C)	*T_p_* (°C)	Δ*T* (°C)	Δ*H_∞_* (J/g)	ΔT*	ΔH*	*CI*
**EP [21]**	2	27.47	130.94	74.30	103.47	353.95	n.a.	n.a.	n.a.
	5	30.67	157.98	88.17	127.31	375.90	n.a.	n.a.	n.a.
	7	33.27	172.55	95.77	139.28	345.48	n.a.	n.a.	n.a.
	10	32.22	183.55	104.41	151.32	387.63	n.a.	n.a.	n.a.
**EP/Zn-Al-CO_3_-HA**	2	27.86	127.39	75.28	99.53	303.06	0.96	0.85	0.82
	5	28.72	150.21	89.14	121.49	322.93	0.95	0.86	0.82
	7	29.65	168.11	95.04	138.46	333.74	0.99	0.96	0.95
	10	33.93	172.55	101.41	138.62	381.53	0.92	0.98	0.98
**EP/Mg-Al-CO_3_-HA**	2	30.29	126.06	74.25	95.77	292.63	0.92	0.83	0.76
	5	32.72	150.61	89.62	117.89	361.07	0.93	0.96	0.89
	7	35.45	149.76	95.00	114.31	309.27	0.82	0.89	0.73
	10	35.94	168.25	102.18	132.31	341.76	0.87	0.88	0.76

n.a.—not applicable (reference measurements).

**Table 4:** The kinetic parameters of neat epoxy and its nanocomposites based on the *Friedman* and *KAS* methods.

Designation	Heating Rate (°C∙min^−1^)	*E_α_* (kJ/mol)	ln(*A*) (1/s)	Mean (1/s)	*m*	Mean	*n*	Mean
***Friedman* method**								
**EP**	2	49.38	15.44	15.52	0.44	0.44	1.40	1.42
	5		15.64		0.44		1.41	
	7		15.48		0.39		1.42	
	10		15.55		0.49		1.46	
**EP/Zn-Al-CO_3_-HA**	2	55.41	17.21	17.23	0.23	0.21	1.35	1.38
	5		17.22		0.19		1.34	
	7		17.23		0.21		1.42	
	10		17.26		0.21		1.42	
**EP/Mg-Al-CO_3_-HA**	2	55.61	17.33	17.28	0.23	0.20	1.37	1.35
	5		17.24		0.19		1.35	
	7		17.29		0.19		1.31	
	10		17.26		0.20		1.36	
***KAS* method**								
**EP**	2	54.37	17.16	17.17	0.39	0.38	1.45	1.47
	5		17.29		0.39		1.45	
	7		17.10		0.33		1.47	
	10		17.13		0.43		1.51	
**EP/Zn-Al-CO_3_-HA**	2	59.94	18.77	18.72	0.18	0.15	1.40	1.42
	5		18.71		0.14		1.38	
	7		18.70		0.15		1.46	
	10		18.70		0.15		1.46	
**EP/Mg-Al-CO_3_-HA**	2	59.05	18.52	18.41	0.20	0.16	1.40	1.38
	5		18.37		0.15		1.38	
	7		18.41		0.14		1.34	
	10		18.36		0.15		1.39	

**Figure A1:**
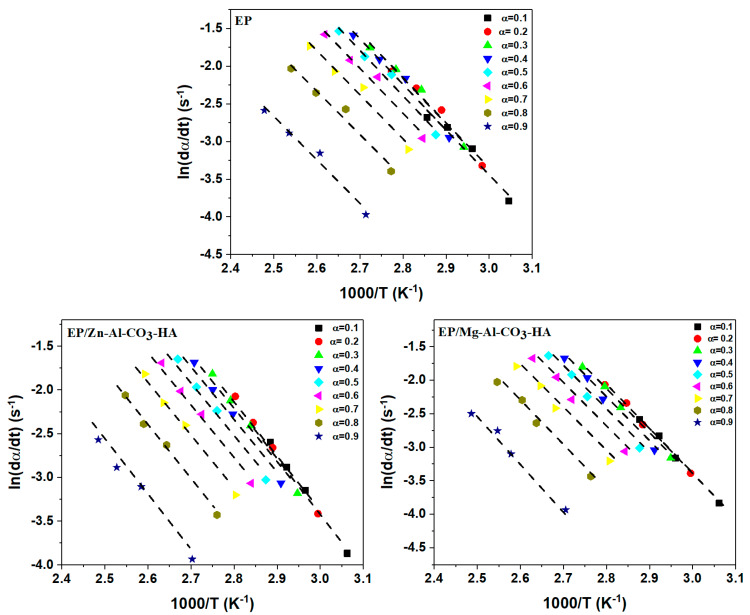
Fitting plots for the neat epoxy and its nanocomposites based on the *Friedman* model.

**Figure A2:**
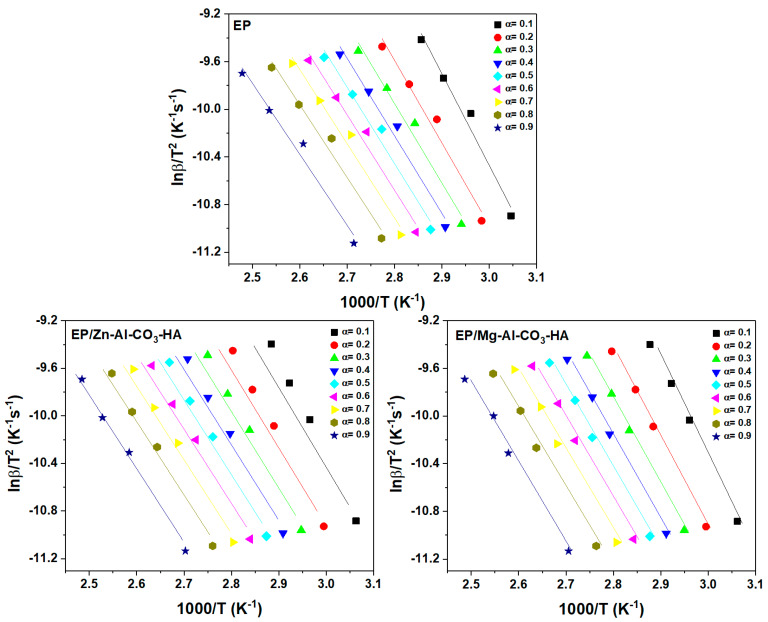
Fitting plots for the neat epoxy and its nanocomposites based on the *KAS* model.

**Figure A3:**
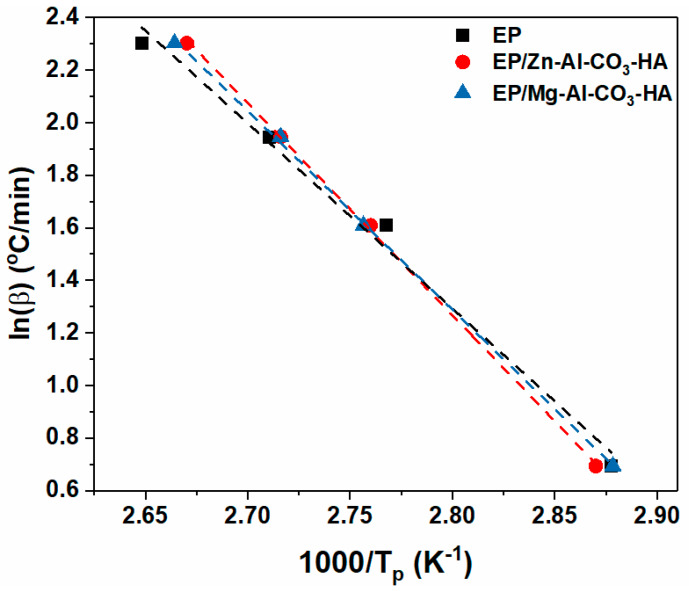
Plots of *ln(β)* vs. 1/*T* for neat epoxy and its prepared nanocomposites based on the *FWO* model.

**Figure A4:**
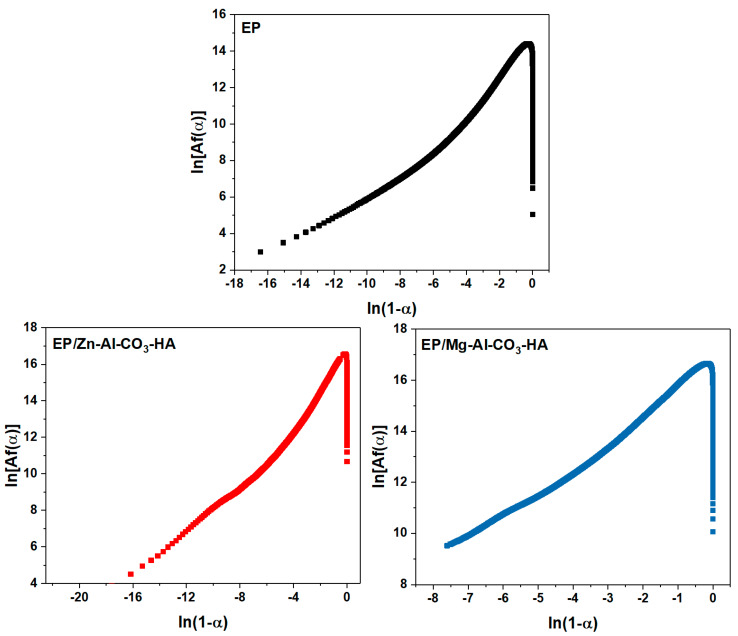
Plots of *ln* [*Af(α)*] vs. *ln(1 − α)* for the samples under a heating rate of 2 °C∙min^−1.^

**Figure A5:**
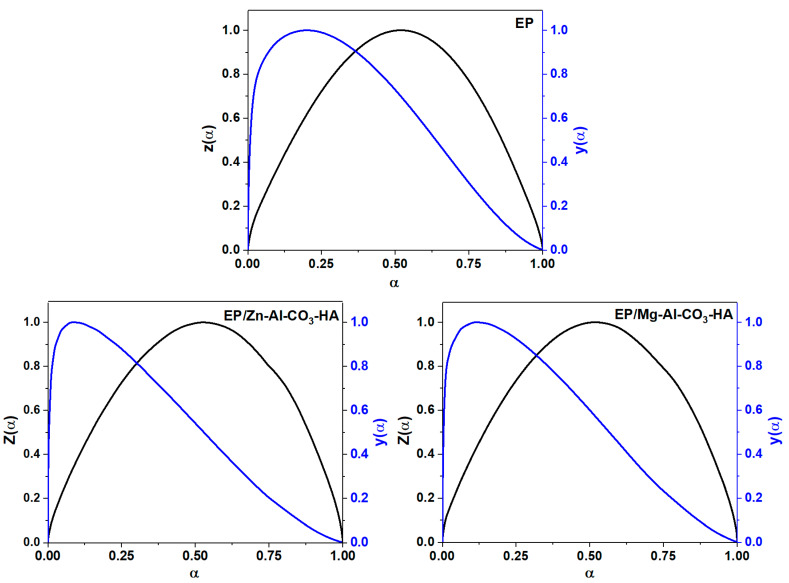
*y(α)* and *Z(α)* curves as a function of α for prepared samples based on the *Málek* model.

**Figure A6:**
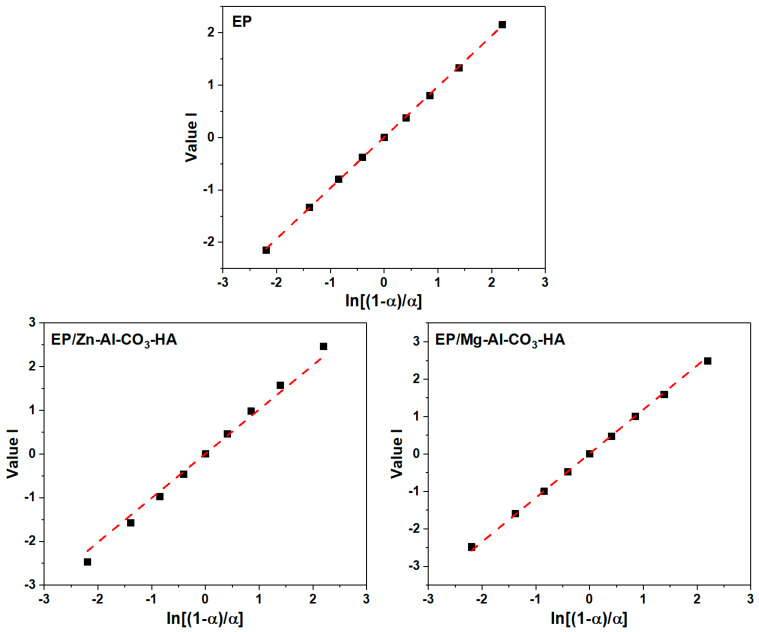
Plots of Value I for the prepared samples.

**Figure A7:**
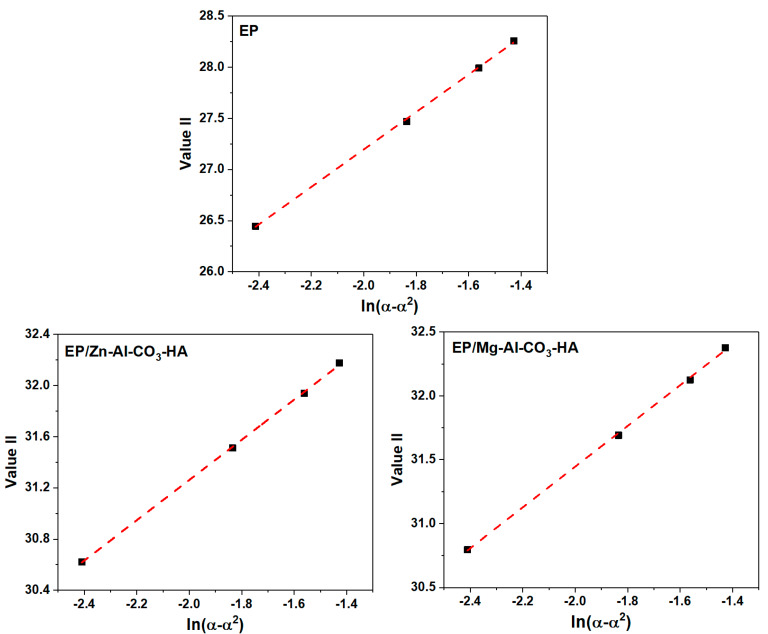
Plots of Value II for the prepared samples.

## Appendix A. Isoconversional Kinetic Methods

### Appendix A.1. Friedman Model

The *Friedman* model is given by [47,48]:

(A1) $$ln\left[\beta_{i}{\left(\frac{d\alpha }{dT}\right)}_{\alpha ,i}\right]=ln\left[f\left(\alpha \right)A_{\alpha }\right]-\frac{E_{a}}{RT_{\alpha ,i}}$$

The plot of $\;ln\left[\beta_{i}{\left(d\alpha /dT\right)}_{\alpha ,i}\right]$ vs. 1/Tα is shown in Figure A1, whose slope gives the value of *E_α_* at each *α*.

### Appendix A.2. KAS Method

The *KAS* method can be described by the following expression [49,50]:

(A2) $$ln\left(\frac{\beta_{i}}{T_{\alpha ,i}^{1.92}}\right)=Const-1.0008\left(\frac{E_{a}}{RT_{\alpha }}\right)$$

By plotting $ln\left(\beta_{i}/T_{\alpha ,i}^{1.92}\right)$ vs. 1/*T_α_*, a straight line is obtained, whose slope gives the *E_α_* (Figure A2).

### Appendix A.3. FWO Model

The *FWO* model is defined by the following equation [51,52]:

(A3) $$ln\left(\beta_{i}\right)=Const-1.052\left(\frac{E_{\alpha }}{RT_{\alpha }}\right)$$

In the *FWO* method, *E_α_* is determined from the slope of *ln(βi)* vs. 1/*T* as shown in Figure A3. In this method, the *E_α_* obtained is independent of the *α*. The values of *E_α_* obtained by the *FWO* method are 55.74 kJ/mol, 63.77 kJ/mol, and 59.87 kJ/mol for neat epoxy, EP/Zn-Al-CO_3_-HA, and EP/Mg-Al-CO_3_-HA, respectively.

## Appendix B. Selection of the Cure Reaction Model

### Appendix B.1. Friedman Model

The *Friedman* method (Equation (A4)) can be used for the determination of the epoxy cure reaction model. The shape of the plot of *ln*[*Af(α)*] vs. *ln(1 − α)* denotes the catalytic or non-catalytic reaction mechanism (Figure A4) [53].

(A4) $$ln\left[Af\left(\alpha \right)\right]=ln\left(\frac{d\alpha }{dt}\right)+\frac{E_{a}}{RT}=lnA+nln\left(1-\alpha \right)$$

For the non-catalytic nth-order cure mechanism, a straight line was obtained by plotting *ln*[*Af(α)*] vs. *ln(1 − α)*, whose slope gives the reaction degree (*n*).

### Appendix B.2. Málek Method

The kinetic model based on the *Málek* method is determined by introducing two functions:

(A5) $$y\left(\alpha \right)={\left(\frac{d\alpha }{dt}\right)}_{\alpha }exp\left(\frac{E_{a}}{RT_{\alpha }}\right)=Af\left(\alpha \right)$$

(A6) $$z\left(\alpha \right)={\left(\frac{d\alpha }{dt}\right)}_{\alpha }T_{\alpha }^{2}$$

In Equation (A5), the constant value of *E_α_* obtained from the *FWO* method was used for the determination of the *y(α)* value. *y(α)* and *z(α)* were normalized as follows to vary between 0 and 1:

(A7) $$y_{n}\left(\alpha \right)=\frac{y\left(\alpha \right)}{max\left[y\left(\alpha \right)\right]}$$

(A8) $$z_{n}\left(\alpha \right)=\frac{z\left(\alpha \right)}{max\left[z\left(\alpha \right)\right]}$$

The maximum values, *max*[*y(α)*] = *α_m_* and *max*[*z(α)*]= *α_p_*, can be found from the following expressions:

(A9) $$f^{\prime }\left(\alpha_{m}\right)=0$$

(A10) $$f^{\prime }\left(\alpha_{p}\right)g\left(\alpha_{p}\right)=-1$$

Based on the *Málek* method, the cure kinetic model is determined using the maximum points of *y(α)* = *(α_m_), z(α)* = *(α_p_^∞^)* as the most important factors and the peak of conversion in the DSC curves (*α_p_*), which is explained by Equations (A7) and (A8) [54,55]. The values of the *Málek* model (*α_m_, α_p_^∞^*, and *α_p_*) for epoxy samples are listed in Table A1. As represented in Table A1, the values of *α_m_* are larger than zero and smaller than *α_p_^∞^*, and the values of *α_p_^∞^* are unequal to 0.632, which represent a two-parameter autocatalytic kinetic model for epoxy systems [56,57]. A comparison between the experimental and theoretical values of *y(α)* and *z(α)* for EP, EP/Zn-Al-CO_3_-HA, and EP/Mg-Al-CO_3_-HA nanocomposites as a function of α is shown in Figure A5.

**Table A1:** The values of *α_p_, α_m_*, and *α_p_^∞^* obtained from the *Málek* model at various heating rates.

Designation	Heating Rate (°C∙min^−1^)	*α_p_^∞^*	*α_m_*	*α_p_*
**EP**	2	0.4934	0.1991	0.5188
	5	0.4892	0.1820	0.5435
	7	0.4808	0.1860	0.5294
	10	0.5606	0.2150	0.5317
**EP/Zn-Al-CO_3_-HA**	2	0.5098	0.0898	0.5279
	5	0.5601	0.0755	0.5320
	7	0.4990	0.0626	0.5229
	10	0.5327	0.0408	0.5300
**EP/Mg-Al-CO_3_-HA**	2	0.4946	0.1161	0.5181
	5	0.5497	0.1032	0.5261
	7	0.6362	0.0974	0.5346
	10	0.5909	0.0914	0.5337

## Appendix C. Determination of the Degree of Reaction

As the autocatalytic cure mechanism for epoxy nanocomposites was determined by the *Friedman* and *Málek* models, the curing kinetics of epoxy nanocomposites can be explained by the following equation [58]:

(A11) $$\frac{d\alpha }{dt}=A\exp(-\frac{E_{\alpha }}{RT})\alpha^{m}{\left(1-\alpha \right)}^{n}$$

The degrees of the autocatalytic and noncatalytic reactions (*m* and *n*) and the frequency factor (*A*) can be calculated by the following equations [59,60]:

(A12) $$Value\;I=ln\left(\frac{d\alpha }{dt}\right)+\frac{E_{a}}{RT}-ln\left[\frac{d\left(1-\alpha \right)}{dt}\right]-\frac{E_{a}}{RT^{\prime }}=\left(n-m\right)ln\left(\frac{1-\alpha }{\alpha }\right)$$

(A13) $$Value\;II=ln\left(\frac{d\alpha }{dt}\right)+\frac{E_{a}}{RT}+ln\left[\frac{d\left(1-\alpha \right)}{dt}\right]+\frac{E_{a}}{RT^{\prime }}=\left(n+m\right)ln\left(\alpha -\alpha^{2}\right)+2lnA$$

The slope of the plot of Value I vs. *ln* [*(1 − α)/α*] (Figure A6) gives the value of (*n − m*), and the slope and intercept of the plot of Value II vs. *ln(α − α^2^)* (Figure A7) gives the values of (*n + m*) and 2*lnA*.

## References

[1] Vahabi H., Jouyandeh M., Cochez M., Khalili R., Vagner C., Ferriol M., Movahedifar E., Ramezanzadeh B., Rostami M., Ranjbar Z. (2018). Short-lasting fire in partially and completely cured epoxy coatings containing expandable graphite and halloysite nanotube additives. Prog. Org. Coat..

[2] Saeb M.R., Najafi F., Bakhshandeh E., Khonakdar H.A., Mostafaiyan M., Simon F., Scheffler C., Mäder E. (2015). Highly curable epoxy/MWCNTs nanocomposites: An effective approach to functionalization of carbon nanotubes. Chem. Eng. J..

[3] Hu Q., Memon H., Qiu Y., Wei Y. (2019). The Failure mechanism of composite stiffener components reinforced with 3D woven fabrics. Materials.

[4] Weil E.D., Levchik S. (2004). A review of current flame retardant systems for epoxy resins. J. Fire Sci..

[5] Jouyandeh M., Jazani O.M., Navarchian A.H., Shabanian M., Vahabi H., Saeb M.R. (2019). Bushy-surface hybrid nanoparticles for developing epoxy superadhesives. Appl. Surf. Sci..

[6] Mohan P. (2013). A critical review: The modification, properties, and applications of epoxy resins. Polym. Plast Tech. Eng..

[7] Bukhtiyarova M. (2019). A review on effect of synthesis conditions on the formation of layered double hydroxides. J. Solid State Chem..

[8] Mishra G., Dash B., Pandey S. (2018). Layered double hydroxides: A brief review from fundamentals to application as evolving biomaterials. Appl. Clay. Sci..

[9] Cao Z., Li B., Sun L., Li L., Xu Z.P., Gu Z. (2020). 2D layered double hydroxide nanoparticles: Recent progress toward preclinical/clinical nanomedicine. Small Methods.

[10] Qu J., Zhang Q., Li X., He X., Song S. (2016). Mechanochemical approaches to synthesize layered double hydroxides: A review. Appl. Clay Sci..

[11] Daud M., Hai A., Banat F., Wazir M.B., Habib M., Bharath G., Al-Harthi M.A. (2019). A review on the recent advances, challenges and future aspect of layered double hydroxides (LDH)–Containing hybrids as promising adsorbents for dyes removal. J. Mol. Liq..

[12] Becker C.M., Gabbardo A.D., Wypych F., Amico S.C. (2011). Mechanical and flame-retardant properties of epoxy/Mg–Al LDH composites. Compos. Part A Appl. Sci. Manuf..

[13] Harun W., Asri R., Alias J., Zulkifli F., Kadirgama K., Ghani S., Shariffuddin J. (2018). A comprehensive review of hydroxyapatite-based coatings adhesion on metallic biomaterials. Ceram. Int..

[14] Ferraz M., Monteiro F., Manuel C. (2004). Hydroxyapatite nanoparticles: A review of preparation methodologies. J. Appl. Biomater. Biom..

[15] Zhao J.-L., Fu T., Han Y., Xu K.-W. (2004). Reinforcing hydroxyapatite/thermosetting epoxy composite with 3-D carbon fiber fabric through RTM processing. Mater. Lett..

[16] Roese P.B., Amico S.C., Kindlein Júnior W. (2009). Thermal and microestructural characterization of epoxy-infiltrated hydroxyapatite composite. Mater. Res..

[17] Jouyandeh M., Rahmati N., Movahedifar E., Hadavand B.S., Karami Z., Ghaffari M., Taheri P., Bakhshandeh E., Vahabi H., Ganjali M.R. (2019). Properties of nano-Fe_3_O_4_ incorporated epoxy coatings from Cure Index perspective. Prog. Org. Coat..

[18] Gao Y., Qiu L., O’Hare D., Wang Q. (2020). Thermal properties and flame-retardant characteristics of layered double hydroxide polymer nanocomposites. Layered Double Hydroxide Polymer Nanocomposites.

[19] Jouyandeh M., Tikhani F., Shabanian M., Movahedi F., Moghari S., Akbari V., Gabrion X., Laheurte P., Vahabi H., Saeb M.R. (2020). Synthesis, characterization, and high potential of 3D metal–organic framework (MOF) nanoparticles for curing with epoxy. J. Alloys Compd..

[20] Jouyandeh M., Karami Z., Jazani O.M., Formela K., Paran S.M.R., Jannesari A., Saeb M.R. (2019). Curing epoxy resin with anhydride in the presence of halloysite nanotubes: The contradictory effects of filler concentration. Prog. Org. Coat..

[21] Karami Z., Jouyandeh M., Ali J.A., Ganjali M.R., Aghazadeh M., Paran S.M.R., Naderi G., Puglia D., Saeb M.R. (2019). Epoxy/layered double hydroxide (LDH) nanocomposites: Synthesis, characterization, and Excellent cure feature of nitrate anion intercalated Zn-Al LDH. Prog. Org. Coat..

[22] Karami Z., Aghazadeh M., Jouyandeh M., Zarrintaj P., Vahabi H., Ganjali M.R., Torre L., Puglia D., Saeb M.R. (2020). Epoxy/Zn-Al-CO_3_ LDH nanocomposites: Curability assessment. Prog. Org. Coat..

[23] Karami Z., Jouyandeh M., Ghiyasi S., Ali J.A., Ganjali M.R., Aghazadeh M., Maadani M., Rallini M., Luzi F., Torre L. (2020). Exploring curing potential of epoxy nanocomposites containing nitrate anion intercalated Mg–Al–LDH with Cure Index. Prog. Org. Coat..

[24] Karami Z., Jouyandeh M., Hamad S.M., Ganjali M.R., Aghazadeh M., Torre L., Puglia D., Saeb M.R. (2019). Curing epoxy with Mg-Al LDH nanoplatelets intercalated with carbonate ion. Prog. Org. Coat..

[25] Wang X., Zhu X., Meng X. (2017). Preparation of a Mg/Al/Fe layered supramolecular compound and application for removal of Cr (VI) from laboratory wastewater. RSC Adv..

[26] Yin H., Cui L., Ai S., Fan H., Zhu L. (2010). Electrochemical determination of bisphenol A at Mg–Al–CO_3_ layered double hydroxide modified glassy carbon electrode. Electrochim. Acta.

[27] Karami Z., Jouyandeh M., Ali J.A., Ganjali M.R., Aghazadeh M., Maadani M., Rallini M., Luzi F., Torre L., Puglia D. (2019). Development of Mg-Zn-Al-CO_3_ ternary LDH and its curability in epoxy/amine system. Prog. Org. Coat..

[28] Mahjoubi F.Z., Khalidi A., Abdennouri M., Barka N. (2017). Zn–Al layered double hydroxides intercalated with carbonate, nitrate, chloride and sulphate ions: Synthesis, characterisation and dye removal properties. J. Taibah. Univ. Sci..

[29] Ma S., Chen Q., Li H., Wang P., Islam S.M., Gu Q., Yang X., Kanatzidis M.G. (2014). Highly selective and efficient heavy metal capture with polysulfide intercalated layered double hydroxides. J. Mater. Chem. A.

[30] Costa D.G., Rocha A.B., Souza W.F., Chiaro S.S.X., Leitão A.A. (2012). Comparative Structural, thermodynamic and electronic analyses of Zn–Al–A^n−^ hydrotalcite-like compounds (A^n−^
*=* Cl^−^, F^−^, Br^−^, OH^−^, CO_3_^2−^ or NO^3−^): An ab initio study. Appl. Clay. Sci..

[31] Liu C., Zhang M., Pan G., Lundehøj L., Nielsen U.G., Shi Y., Hansen H.C.B. (2019). Phosphate capture by ultrathin MgAl layered double hydroxide nanoparticles. Appl. Clay. Sci..

[32] Theiss F.L., Ayoko G.A., Frost R.L. (2013). Thermogravimetric analysis of selected layered double hydroxides. J. Therm. Anal. Calorim..

[33] Jouyandeh M., Karami Z., Ali J.A., Karimzadeh I., Aghazadeh M., Laoutid F., Vahabi H., Saeb M.R., Ganjali M.R., Dubois P. (2019). Curing epoxy with polyethylene glycol (PEG) surface-functionalized Ni_x_Fe_3−x_O_4_ magnetic nanoparticles. Prog. Org. Coat..

[34] Jouyandeh M., Hamad S.M., Karimzadeh I., Aghazadeh M., Karami Z., Akbari V., Shammiry F., Formela K., Saeb M.R., Ranjbar Z. (2019). Unconditionally blue: Curing epoxy with polyethylene glycol (PEG) surface-functionalized Zn_x_Fe_3−x_O_4_ magnetic nanoparticles. Prog. Org. Coat..

[35] Karami Z., Jouyandeh M., Ali J.A., Ganjali M.R., Aghazadeh M., Maadani M., Rallini M., Luzi F., Torre L., Puglia D. (2019). Cure Index for labeling curing potential of epoxy/LDH nanocomposites: A case study on nitrate anion intercalated Ni-Al-LDH. Prog. Org. Coat..

[36] Saeb M.R., Nonahal M., Rastin H., Shabanian M., Ghaffari M., Bahlakeh G., Ghiyasi S., Khonakdar H.A., Goodarzi V., Vijayan P.P. (2017). Calorimetric analysis and molecular dynamics simulation of cure kinetics of epoxy/chitosan-modified Fe_3_O_4_ nanocomposites. Prog. Org. Coat..

[37] Tikhani F., Moghari S., Jouyandeh M., Laoutid F., Vahabi H., Saeb M.R., Dubois P. (2020). Curing kinetics and thermal stability of epoxy composites containing newly obtained nano-scale aluminum hypophosphite (AlPO_2_). Polymers.

[38] Jouyandeh M., Karami Z., Hamad S.M., Ganjali M.R., Akbari V., Vahabi H., Kim S.-J., Zarrintaj P., Saeb M.R. (2019). Nonisothermal cure kinetics of epoxy/Zn_x_Fe_3−x_O_4_ nanocomposites. Prog. Org. Coat..

[39] Jouyandeh M., Ganjali M.R., Ali J.A., Aghazadeh M., Karimzadeh I., Formela K., Colom X., Cañavate J., Saeb M.R. (2019). Curing epoxy with ethylenediaminetetraacetic acid (EDTA) surface-functionalized Co_x_Fe_3−x_O_4_ magnetic nanoparticles. Prog. Org. Coat..

[40] Vyazovkin S., Sbirrazzuoli N. (1999). Isoconversional method to explore the mechanism and kinetics of multi-step epoxy cures. Macromol. Rapid Commun..

[41] Vyazovkin S. (2001). Modification of the integral isoconversional method to account for variation in the activation energy. J. Comput. Chem..

[42] Miura K. (1995). A new and simple method to estimate f (E) and k_0_ (E) in the distributed activation energy model from three sets of experimental data. Energy Fuels.

[43] Mashouf Roudsari G., Mohanty A.K., Misra M. (2014). Study of the curing kinetics of epoxy resins with biobased hardener and epoxidized soybean oil. ACS Sustain. Chem. Eng..

[44] Jouyandeh M., Paran S.M.R., Shabanian M., Ghiyasi S., Vahabi H., Badawi M., Formela K., Puglia D., Saeb M.R. (2018). Curing behavior of epoxy/Fe_3_O_4_ nanocomposites: A comparison between the effects of bare Fe_3_O_4_, Fe_3_O_4_/SiO_2_/chitosan and Fe_3_O_4_/SiO_2_/chitosan/imide/phenylalanine-modified nanofillers. Prog. Org. Coat..

[45] Jouyandeh M., Jazani O.M., Navarchian A.H., Shabanian M., Vahabi H., Saeb M.R. (2018). Surface engineering of nanoparticles with macromolecules for epoxy curing: Development of super-reactive nitrogen-rich nanosilica through surface chemistry manipulation. Appl. Surf. Sci..

[46] Jouyandeh M., Paran S.M.R., Khadem S.S.M., Ganjali M.R., Akbari V., Vahabi H., Saeb M.R. (2020). Nonisothermal cure kinetics of epoxy/Mn_x_Fe_3−x_O_4_ nanocomposites. Prog. Org. Coat..

[47] Vyazovkin S., Burnham A.K., Criado J.M., Pérez-Maqueda L.A., Popescu C., Sbirrazzuoli N. (2011). ICTAC Kinetics Committee recommendations for performing kinetic computations on thermal analysis data. Thermochim. Acta.

[48] Wang Z., Liu L., Zhang J., Cao L., Dong H., Zhang C., Xu X., Zhu M., Li J. (2019). Optimizing curing process of graphene oxide/waterborne epoxy blends by curing kinetics simulation considering the coupling of heat conduction and curing reaction. Thermochim. Acta.

[49] Kissinger H.E. (1957). Reaction kinetics in differential thermal analysis. Anal. Chem..

[50] Akahira T., Sunose T. (1971). Res. Report Chiba Inst. Technol. Sci. Technol..

[51] Ozawa T. (1970). Kinetic analysis of derivative curves in thermal analysis. J. Therm. Anal. Calorim..

[52] Vyazovkin S. (2006). Model-free kinetics. J. Therm. Anal. Calorim..

[53] Sbirrazzuoli N. (2007). Is the Friedman method applicable to transformations with temperature dependent reaction heat?. Macromol. Chem. Phys..

[54] Montserrat S., Málek J. (1993). A kinetic analysis of the curing reaction of an epoxy resin. Thermochim. Acta.

[55] Tripathi G., Srivastava D. (2009). Cure kinetics of ternary blends of epoxy resins studied by nonisothermal DSC data. J. Appl. Polym. Sci..

[56] Roşu D., Caşcaval C., Mustatǎ F., Ciobanu C. (2002). Cure kinetics of epoxy resins studied by non-isothermal DSC data. Thermochim. Acta.

[57] Kumar S., Samal S.K., Mohanty S., Nayak S.K. (2017). Study of curing kinetics of anhydride cured petroleum-based (DGEBA) epoxy resin and renewable resource based epoxidized soybean oil (ESO) systems catalyzed by 2-methylimidazole. Thermochim. Acta.

[58] Luo X., Yu X., Ma Y., Naito K., Zhang Q. (2018). Preparation and cure kinetics of epoxy with nanodiamond modified with liquid crystalline epoxy. Thermochim. Acta.

[59] Zhou T., Gu M., Jin Y., Wang J. (2005). Studying on the curing kinetics of a DGEBA/EMI-2, 4/nano-sized carborundum system with two curing kinetic methods. Polymer.

[60] Li L., Zeng Z., Zou H., Liang M. (2015). Curing characteristics of an epoxy resin in the presence of functional graphite oxide with amine-rich surface. Thermochim. Acta.

